# Personal

**Published:** 1891-04

**Authors:** 


### Personal.

Dr. Edward N. Brush, formerly of Buffalo, and at one time editor of this Journal, but more recently in charge of the male department of the Pennsylvania Hospital for the Insane, has recently been appointed medical superintendent of the Sheppard Asylum in Baltimore. The trustees of the Sheppard Asylum have secured an experienced and able manager, as well as an accomplished alienist, and we congratulate all the parties in interest.

---

Dr. Roswell Park, of Buffalo, has been invited by a number of physicians in this city to repeat here his Mütter Lectures on Selected Topics in Modern Surgical Pathology. The first lecture of the course was given in the Buffalo Medical College, Saturday evening, March 21, 1891. The others will be delivered Saturday evenings, March 28th, and April 4th, at 8.15 o'clock. All interested are invited to attend.

